# Alteration in sperm mitochondrial membrane potential and antioxidant biomarkers in summer adversely affects Hampshire-Ghungroo crossbred boar semen fertility in sub-tropical climate

**DOI:** 10.3389/fvets.2025.1562988

**Published:** 2025-04-17

**Authors:** Mahak Singh, Apanai Celina, Rahul Katiyar, Sourabh Deori, Ashwani Singh, Vinay Singh, G. D. Singh, J. S. Rajoriya, H. Kalita, V. K. Mishra

**Affiliations:** ^1^Animal Reproduction Laboratory, ICAR Research Complex for NEH Region, Nagaland Centre, Medziphema, Nagaland, India; ^2^Division of Animal Health and Fisheries Science, ICAR Research Complex for NEH Region, Umiam, Meghalaya, India; ^3^College of Veterinary Sciences, Guru Angad Dev Veterinary and Animal Sciences University, Ludhiana, Punjab, India; ^4^ICAR Research Complex for NEH Region, Tripura Centre, Lembucherra, Tripura, India; ^5^Department of Veterinary Clinical Complex, Bihar Veterinary College, Patna, India; ^6^Department of Veterinary Gynaecology and Obstetrics, NDVSU-College of Veterinary Science and Animal Husbandry, Rewa, India; ^7^ICAR Research Complex for NEH Region, Umiam, Meghalaya, India; ^8^Department of Veterinary Parasitology, Lala Lajpat Rai University of Veterinary and Animal Sciences, Hisar, Haryana, India

**Keywords:** Hampshire–Ghungroo boar, heat stress, antioxidant biomarkers, *in vivo* fertility, subtropical

## Abstract

In temperate regions, boars experience environmental heat stress due to the subtropical climate, leading to their semen quality and fertility being severely compromised compared to indigenous breeds. Considering the above effect, the present study aimed to evaluate the effect of season on semen quality, seminal plasma antioxidant status, and *in vivo* fertility of crossbred boars of exotic (50%) and indigenous inheritance in a subtropical climate. A total of 14 Hampshire–Ghungroo crossbred boars were used for this investigation, which took place in both summer and winter. Sperm characteristics, namely motility, viability, abnormality, acrosomal integrity, and the hypo-osmotic swelling test (HOST) results, and mitochondrial membrane potential (MMP) were evaluated. Sperm kinematics parameters were assessed using computer-assisted semen analysis (CASA). Antioxidant biomarkers (glutathione peroxidase, GPx; catalase, CAT; and total antioxidant capacity, TAC) and lipid peroxidation (malondialdehyde, MDA) were analyzed in boars’ seminal plasma. The summer season had a significant (*p* < 0.01) negative impact on reaction time and false mounts, whereas semen volume and sperm concentration were significantly (*p* < 0.01) higher in the winter season. Similarly, sperm abnormalities were significantly (*p* < 0.01) lower in the winter season. In the winter, sperm quality parameters, namely total motility, progressive motility, viability, acrosomal integrity, and HOST reactivity, were significantly (*p* < 0.01) improved. However, during the summer, sperm MMP was significantly (*p* < 0.01) lower in fresh samples and after 72 h of storage. Season had a significant (*p* < 0.05) effect on the following sperm kinematics parameters: average path velocity, straight-line velocity, curve linear velocity, amplitude of lateral head displacement, and beat cross frequency. Semen characteristics were significantly (*p* < 0.01) improved in winter after 72 h of cold storage compared with those in summer. The summer season had a significant effect (*p* < 0.01) on seminal plasma antioxidant biomarkers (TAC, MDA, CAT, and GPx). Furthermore, the farrowing rate was significantly (*p* < 0.05) higher in the winter season. In conclusion, our results showed that the low MMP of boar sperm and the downregulation of seminal plasma antioxidant biomarkers in summer lead to poor semen quality and poor fertility in Hampshire–Ghungroo crossbred boars in a subtropical climate. To alleviate the heat-stress-induced poor sperm fertility in boars and to optimize the fertility of boars during summer in subtropics, there is a need for scientific interventions in terms of genetics [less exotic inheritance (below 50%)], nutrition, and management.

## Introduction

Boars are sensitive to heat stress because of their high metabolism, poorly developed thermoregulatory system, lack of functional sweat glands, and huge deposits of subcutaneous fats that impair the loss of heat by sweating (1). The thermoneutral zone for adult boars is between 20 and 25°C, and beyond this temperature range, they experience heat stress. With climate change and the increase in summer temperature, the welfare, health, and reproduction of animals are compromised (2). The microclimate in animal farms is increasingly affected by high ambient temperature (3). In tropics and subtropics, temperature and humidity are higher during summer than in regions with a temperate climate. This finding leads to a high temperature humidity index (THI), which is a significant heat stressor in boars. Furthermore, reliance on a few temperate boar breeds for breeding and commercial purposes in tropical and subtropical regions may lead to suboptimal performance of these breeds due to high THI-induced heat stress.

In boars, heat stress causes changes in their physiological and behavioral characteristics to maintain homeothermia, and this finding adversely affects their reproductive function and fertility (4–6). Alterations in photoperiod and temperature affect their semen quality and fertility (7). Their reproductive behavior is season dependent, and this trait is inherited from the European wild boar (*Sus scrofa ferus*) (7). Previous studies have documented the effect of temperature and season on semen quality and fertility of boars (8–10). In a hot and humid climate, environment-induced heat stress reduces their semen quality and fertility (6, 11). The season-dependent fertility of boars in hot and humid tropical and subtropical environments limits the use of high-indexed breeding boars in artificial insemination (AI) breeding programs because in these climate settings, high temperature and humidity lead to heat stress, which results in decreased sperm functional competence and fertility in boars (7, 12–15). A previous study has shown that heat-stress-induced cell damage is more pronounced during spermatogenesis and in germ cells (16). Furthermore, heat stress causes hormonal imbalances, including changes in corticosteroid and cytokine levels, along with the downregulation of the steroidogenic acute regulatory (StAR) protein and the StAR gene (17). Heat stress affects the hypothalamus–pituitary–gonadal axis and leads to a decrease in testosterone concentration in blood and in testes, which is critical for spermatogenesis and maintaining testicular integrity (18). In addition, ambient heat stress increases oxidative stress, germ cell death, insult to sperm DNA, and testicular damage (5). During summer, the low semen output with a compromised sperm quality leads to a decrease in boar fertility (6, 14). Moreover, boar sperm have a unique membrane composition with a low cholesterol-to-phospholipids ratio, which makes it susceptible to oxidative damage during liquid storage (6). Besides, the antioxidant ambience of boar semen is compromised during cold storage in summer due to an increase in the production of reactive oxygen species (ROS). Because of these factors, boar sperm are more vulnerable to membrane lipid peroxidation and apoptosis during cold storage (6, 19).

Temperate boar breeds have been extensively used for breeding and commercial purposes in regions with a subtropical climate to harness their high growth potential. During this process, they have been exposed to the harsh climatic conditions of tropical and subtropical regions, which adversely affect their fertility. Since the effects of heat stress are more pronounced in pure exotic boar breeds, crossbreeding with indigenous boar breeds has been suggested as an alternative strategy to reduce climate-induced heat stress. Although numerous studies in the literature have recorded the effects of different seasons on boar semen quality, most of them were conducted in regions with a temperate climate and on breeds prevalent there. Scant literature is available on sperm quality, antioxidant markers, and *in vivo* fertility of boars during different seasons in a hot and humid subtropical climate. Moreover, only a few *in vitro* studies are available on crossbred boars of tropical and temperate inheritance under subtropical climatic conditions. Hence, the present study aimed (i) to assess the effect of summer and winter seasons on sperm quality parameters (SQPs) and *in vivo* fertility of Hampshire–Ghungroo crossbred boars in a subtropical climate and (ii) to evaluate seminal plasma antioxidant biomarkers and lipid peroxidation in the summer and winter seasons.

## Materials and methods

### Ethical approval

The ethical approval for this study was granted by the Institutional Animal Ethics Committee registered with the Committee for Control and Supervision of Experiments on Animals (CCSEA) and the Institutional Research Committee (IXX18384) of the ICAR Research Complex for North Eastern Hill Region, Umiam, Meghalaya. The research farm was registered with (2,100/GO/RBi/L/20/CPCSEA) and approved for research on swine by the Committee for the Purpose of Control and Supervision of Experiments on Animals in accordance with the national guidelines.

### Experiment location

The experiment was carried out at the Pig Research Farm, ICAR Research Complex for North Eastern Hill Region, Nagaland Centre, India. The farm is located at an altitude of 281 m above the mean sea level, a latitude of 25°45′ N, and a longitude of 93°50′ E. The study was carried out in summer (June to July) and winter seasons (December to January). The experimental site has a hot and humid subtropical climate, with annual rainfall varying from 1,500 to 2,000 mm. The winter season is mild with temperate weather, whereas the summer season is hot and humid. An automated weather station of Gramin Krishi Mausam Seva, located near the Pig Research Farm, recorded temperature and humidity data. The THI was determined using the following formula (6, 20):


$$THI=\left(0.8\;x\;T\;\left({}^{\circ}C\right)+\left(RH\;\left(\%\right)/100\right)\;x\;\left(T\;\left({}^{\circ}C\right)-14.4\right)+46.4\right)$$

where T represents temperature in °C and RH represents relative humidity.

**Figure 1:**
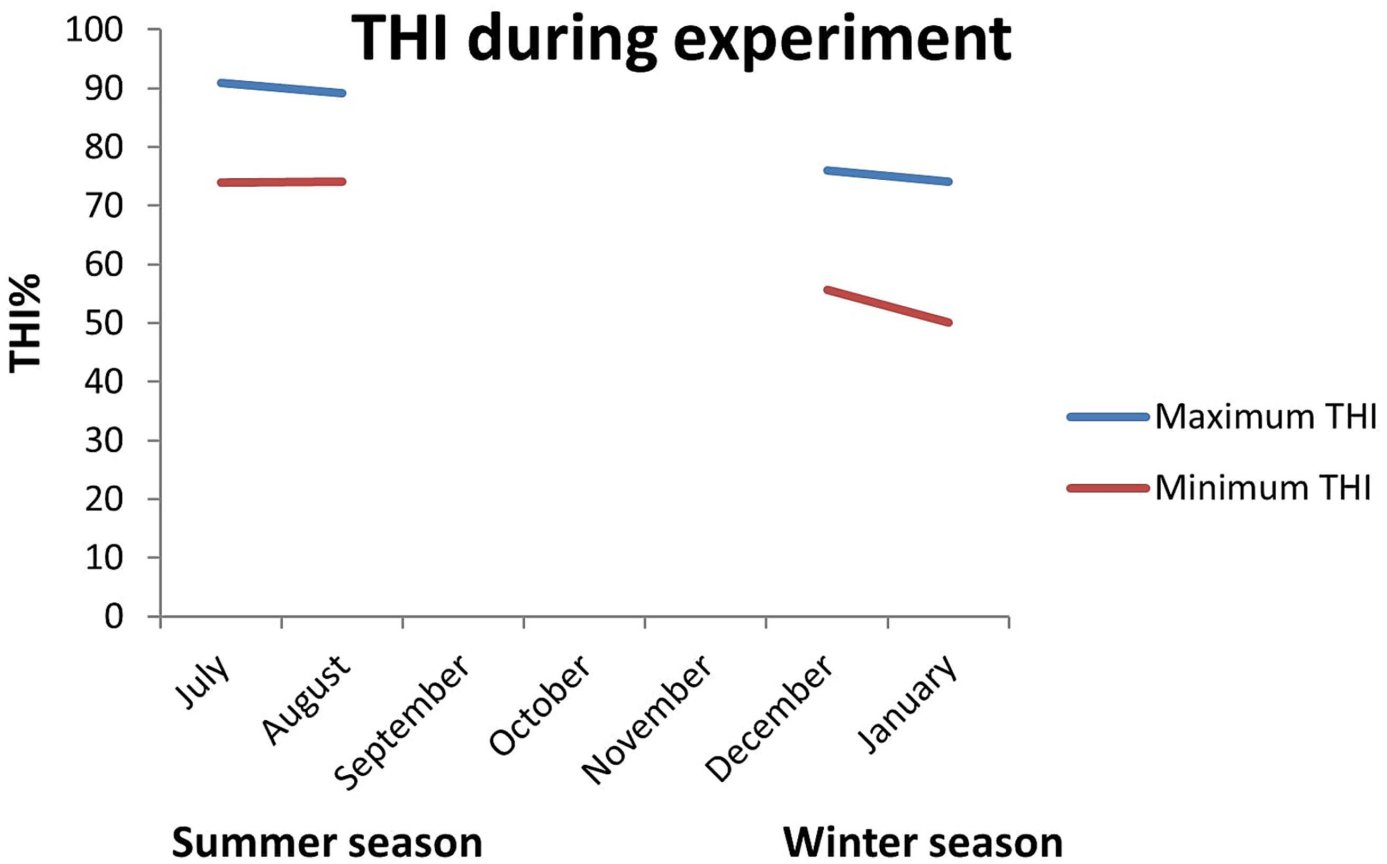
The maximum and minimum temperature humidity index during summer and winter seasons at the experiment station (mean ± SEM).

During the summer season, the maximum and minimum temperature were 33.6°C and 24.9°C, respectively, and the maximum and minimum relative humidity were 92 and 71%, respectively. However, in the winter season, the maximum and minimum temperature were 24.6°C and 9.0°C, respectively, and the maximum and minimum relative humidity were 96 and 62%, respectively. Similarly, in summer, the maximum and minimum THI were 90.95 and 74, respectively, whereas, in winter, they were 75.92 and 50.07, respectively (Figure 1). THI is categorized into heat stress thresholds as follows: THI values between 74 and 78 suggest mild heat stress, those between 78 and 82 indicate moderate heat stress, and values of 82 and beyond indicate severe heat stress (21). A THI value of less than 74 indicates a pleasant environment. As the experimental location is a subtropical region, variations in photoperiod between the seasons were minimum and thus were not taken into account.

### Animals

In this investigation, 14 crossbred boars (50% Hampshire and 50% Ghungroo), ranging in age from 18 to 30 months, were used. The same boars were used in both summer and winter seasons. They were kept in separate concrete pens, each measuring 9 m^2^, with an open space. Since there were no facilities for temperature control, the weather inside and outside the enclosure was the same. Electrical fans were installed in boar pens and used during the summer season, but no sprinklers were used. Uniform management procedures were followed at the study farm, including consistent lighting, housing, and food.

### Experimental design

**Figure 2:**
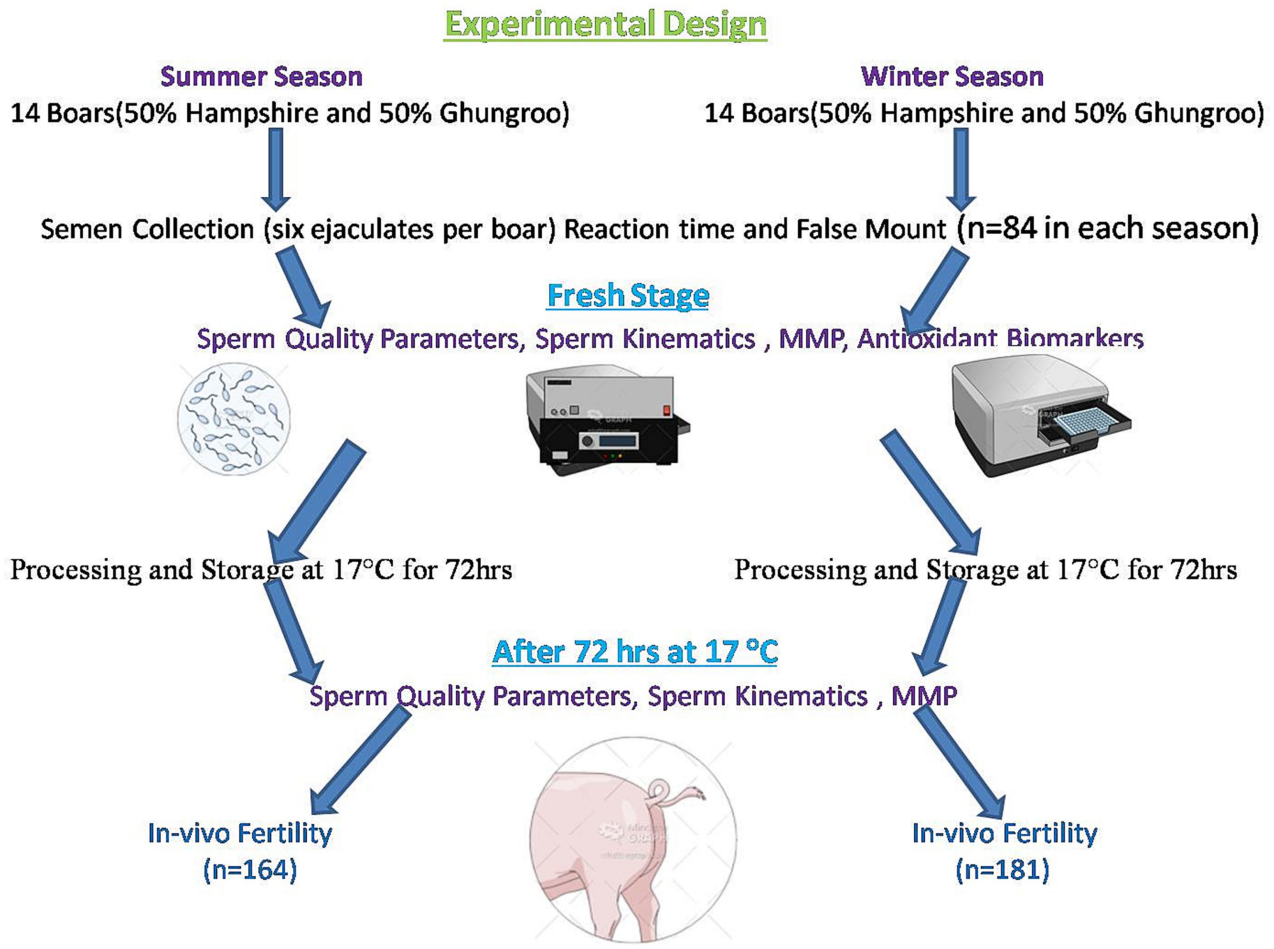
Graphical representation of the experimental design.

A graphical representation of the experimental design is presented in Figure 2. All of the 14 boars were of the same genetic composition (50% Hampshire and 50% Ghungroo). Their diet comprised a maize and groundnut cake, with a daily feed limit of 3.0 kg (6). Food was served in equal proportions in the morning and the evening. Based on the proposal of the National Research Council, the nutritional needs were determined. The boars were given *ad libitum* access to drinking water. During a period of 6 weeks, semen samples were collected every week from each boar, totaling six samples per animal.

### Sample collection

As mentioned earlier, semen samples were collected once a week from each boar using the gloved-hand technique for 6 weeks. Six ejaculates were collected from each boar during both summer and winter, resulting in a total of 168 semen samples in both seasons. A prewarmed thermos flask was used to collect semen ejaculates. During semen collection, only sperm-rich middle fractions of the ejaculates were collected, and they were sent to the laboratory immediately after collection (in 15 min). In the laboratory, the samples were transferred to a sterile glass flask and kept in a water bath at 37 °C. Then, they were examined for SQPs and extended in a PRIMXcell (IMV, France) medium kept at 37 °C. The semen samples were then diluted with PRIMXcell so that each 80 mL semen pouch (GTB Bag Manual, IMV, France) contained 3 billion motile spermatozoa. After filling the GTB bags, they were sealed and kept in a biochemical oxygen demand (BOD) incubator (Innovative Technologies, India) at 17 °C for 72 h. After 72 h of cold storage, the semen pouches were taken out, warmed at 37 °C for 2 min, and analyzed for sperm functional characteristics.

### Reaction time and false mount

The time interval from the boar entering the collection room to its first attempt to mount the dummy was recorded as the reaction time (in minutes) (6). The number of times the boar mounted on a dummy but dismounted before semen ejaculation (mounts with no ejaculation) was considered false mounts (numbers).

### Semen quality analysis

A graduated cylinder was used to measure semen volume (mL). SQPs, namely motility, viability, abnormality, acrosomal integrity, and hypo-osmotic swelling test (HOST) reactivity, were assessed using a phase-contrast microscope (TempStar, India) at the time of collection and after 72 h of cold storage.

### Sperm concentration

A hemocytometer was used to measure sperm concentration (millions per milliliter) (22). Briefly, 1% PBS-buffered formalin was added to the semen sample. Then, the hemocytometer was charged with the semen and allowed to settle. Sperm heads in five squares were counted in each chamber under a microscope (TempStar, India) with 400 × magnification, and the counts on both sides were averaged to obtain the final concentration.

### Assessment of sperm viability

The differential staining technique was used to determine sperm viability (23). In this method, a thin semen smear with diluted semen was prepared on a warmed glass slide. After drying the smear, a minimum of 200 spermatozoa were counted under oil immersion (1000×). Sperm were considered dead if a pinkish (eosin) color was present; no stain color in the sperm head meant that the sperm were live. To determine sperm abnormality, the slides were examined for abnormal heads, abnormal tails, abnormal mid-pieces, detached heads, coiled tails, and the presence of proximal cytoplasmic droplets (24).

### Assessment of sperm acrosome integrity

Giemsa staining was used to assess sperm acrosome integrity under oil immersion (25). Briefly, a thin semen smear was prepared on a glass slide and fixed with Hancock fixative. In each slide, 200 spermatozoa were counted for acrosomal integrity, and acrosomes were considered intact if the entire acrosomal cap was present.

### Assessment of sperm plasma membrane integrity

The HOST was used to assess sperm plasma membrane integrity (6). In this method, a hypo-osmotic solution (150 mOsm/L) was prepared using 7.35 g sodium citrate and 13.5 g fructose in 1 L double-distilled water. Then, 900 μL of the HOST solution was mixed with 100 μL semen sample and incubated at 37°C. After 45 min of incubation, eosin stain was added to the solution to enhance sperm visibility. A thin smear was prepared on a prewarmed glass slide and air-dried. After air drying, the slides were examined using a phase-contrast microscope under 400 × magnification. A minimum of 200 sperm cells were counted for the HOST reaction in five different microscopic fields. Bent and curled tails indicated that sperm cells had an intact plasma membrane, which were counted as HOST-reactive spermatozoa.

### Assessment of sperm kinematics

Sperm kinematics parameters were assessed using computer-assisted semen analysis (CASA) (6). The following kinematics parameters were assessed: average path velocity (VAP), straight-line velocity (VSL), curve linear velocity (VCL), amplitude of lateral head displacement (ALH), beat cross frequency (BCF), straightness (STR), and linearity (LIN). In this approach, the Hamilton Thorne Sperm Analyser (HTM-IVOS, version IVOS 11, Hamilton Thorne Research, USA) was used. Total motility and progressive motility of sperm were also recorded. The CASA thresholds were set as follows: temperature of analysis (°C): 37; chamber type: Leja 4; frame rate (Hz): 60; fields acquired: 10; minimum static contrast: 35; number of frames: 30; STR (%): 70; minimum cell size (pixels): 5; VAP cutoff (mm/s): 30; VSL cutoff (mm/s): 15; progressive minimum VAP (mm/s): 50; magnification: 1.89; and cell intensity: 80. For CASA, semen was diluted in a PRIMXcell medium maintained at 37°C. Thereafter, 4 μL of diluted semen was kept on a prewarmed (37°C) Leja slide chamber (Leja 4, IMV, France). Then, semen was allowed to settle on a heating plate (38°C), and sperm kinematics were analyzed. Five microscopic fields were analyzed for each sample, in duplicate, and the results were calculated based on the visualization of 500 cells per sample.

### Assessment of sperm mitochondrial membrane potential

The MMP of sperm cells was recorded using a mitochondrial membrane potential (MMP) kit (MAK160, Sigma-Aldrich) in accordance with the manufacturer’s protocol. This kit uses JC-10, a superior alternative to JC-1, for determining the loss of MMP in cells. JC-10 is a cationic, lipophilic dye that is concentrated and forms reversible red-fluorescent JC-10 aggregates (lex = 540/lem = 590 nm) in the mitochondria of cells with a polarized mitochondrial membrane. In apoptotic cells, MMP collapse leads to the failure to retain JC-10 in the mitochondria, and the dye returns to its monomeric, green-fluorescent form (lex = 490/lem = 525 nm). Sperm cells were observed under a fluorescent microscope (TempStar, India) to count at least 200 cells.

### Seminal plasma antioxidant biomarkers

Seminal plasma antioxidant biomarkers (GPx, TAC, and CAT) and lipid peroxidation (malondialdehyde, MDA) were analyzed in summer and winter. Immediately after semen collection, a small portion of the ejaculate was centrifuged at 1000 × g/min for 15 min, and seminal plasma was aspirated in another vial. The aspirated seminal plasma was stored in 2.0-mL Eppendorf (EP) tubes at −20°C for future analysis. Absorbance of the samples was measured using a ThermoScientific Multiskan GO Microplate Spectrophotometer, USA (India). For antioxidant biomarkers and lipid peroxidation, the samples were analyzed in duplicate.

### Glutathione peroxidase (GPx) assay

Cayman’s Glutathione Peroxidase Assay Kit (catalog no. 703102, Cayman Chemical Co., USA) was used to measure seminal plasma GPx activity, according to the manufacturer’s protocol. The results were expressed in nmol/min/mL. The intra-assay and inter-assay coefficients of variation were 5.7% (*n* = 77) and 7.2% (*n* = 77), respectively.

### Malondialdehyde (MDA) assay

Caymen’s Thiobarbituric Acid Reactive Substances (TBARS) Assay Kit (catalog no. 10009055, Cayman Chemical Co., USA) was used to analyze MDA concentration, in accordance with the manufacturer’s guidelines. MDA concentration was expressed in nmol/mL. The intra-assay and inter-assay coefficients of variation were 5.5% (*n* = 10) and 5.9% (*n* = 8), respectively.

### Total antioxidant capacity (TAC) assay

Caymen’s Antioxidant Assay Kit (catalog no. 709001, Cayman Chemical Co., USA) was used to assess the seminal plasma total antioxidant capacity (TAC) level (mM), in accordance with the manufacturer’s guidelines. Total antioxidant concentration was expressed in mmol/L. The intra-assay and inter-assay coefficients of variation were 3.4% (*n* = 84) and 3% (*n* = 20), respectively.

### Catalase (CAT) activity

Caymen’s Catalase Assay Kit (catalog no. 707002, Cayman Chemical Co., USA) was used to analyze catalase (CAT) activity in boar’s seminal plasma, in accordance with the the manufacturer’s instructions. CAT activity was expressed in nmol/min/mL. The intra-assay and inter-assay coefficients of variation were 3.8% (*n* = 45) and 9.9% (*n* = 45), respectively.

### Assessment of *in vivo* fertility

To evaluate *in vivo* fertility, AI was performed twice at 12-h intervals in pluriparous sows (*n* = 164 during the summer and *n* = 181 during the winter). The sows were selected based on the body condition score (BCS ≥ 3) and those that came into heat within 10 days of weaning. A golden gilt catheter (IMV) was used for AI in sows. AI was performed 24 h after estrus detection and repeated after 12 h. The farrowing rate (FR) was calculated as the ratio of the number of sows who farrowed to the number of sows who were inseminated. The total number of live piglets farrowed per sow was considered litter size at birth (LSB). Weaning was carried out at 42 days of age, and accordingly, litter size at weaning (LSW) was also recorded.

### Statistical analysis

The data were analyzed for normal distribution using the Shapiro–Wilk test. Homogeneity of variance of data was analyzed using Levene’s test. The means of the two groups were analyzed using an independent-sample t-test to determine the significant difference between them. The Chi-squared test was used to compare the FR of the two seasons. The results were presented as mean ± SEM. The differences were considered significant at a *p*-value of <0.05. Statistical analysis was performed using IBM Statistical Package for the Social Sciences (SPSS), version 27.

## Results

### Boars’ sexual behavior and semen characteristics at the fresh stage

**Figure 3:**
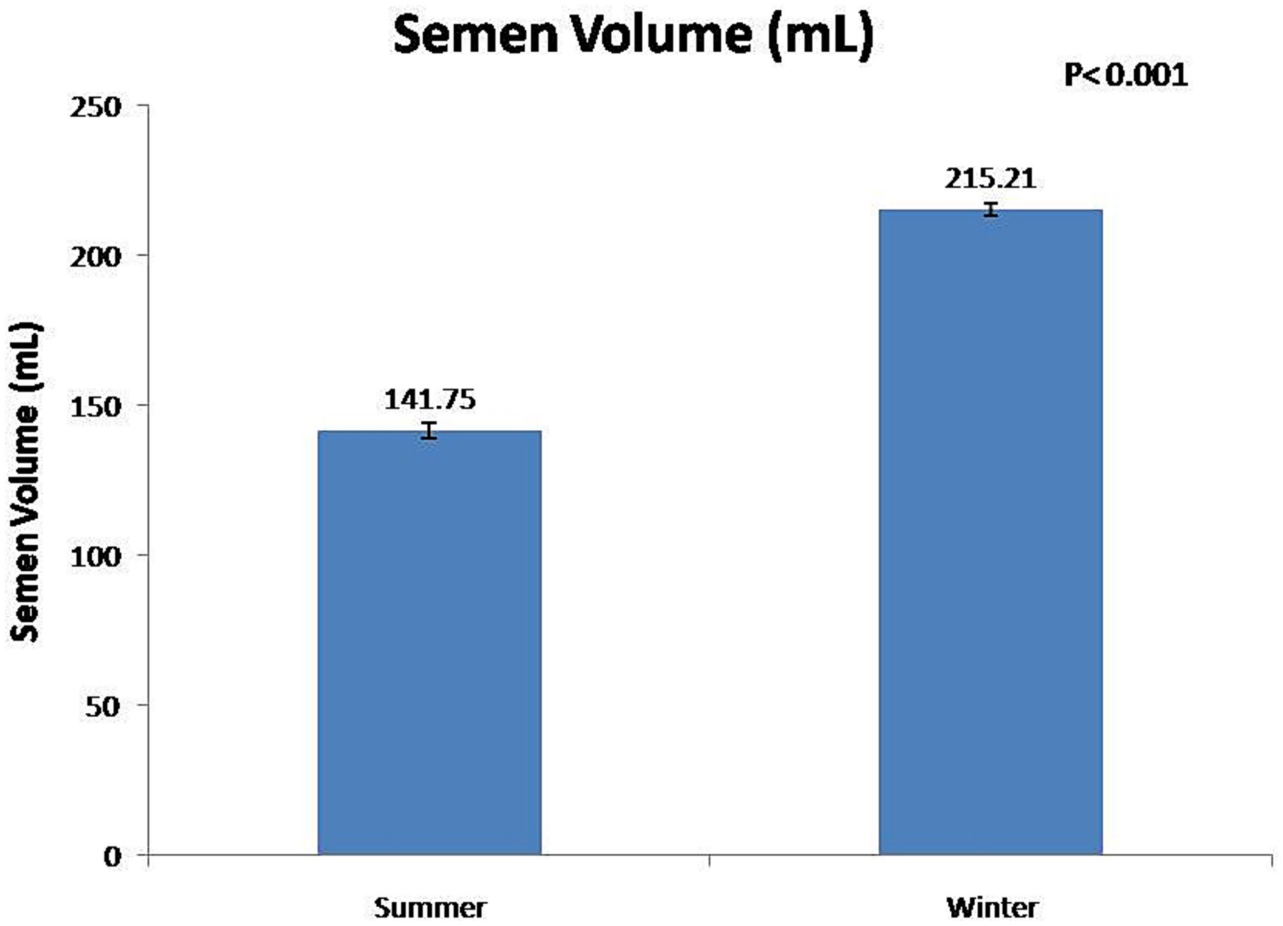
Effect of season on boar semen volume.

**Figure 4:**
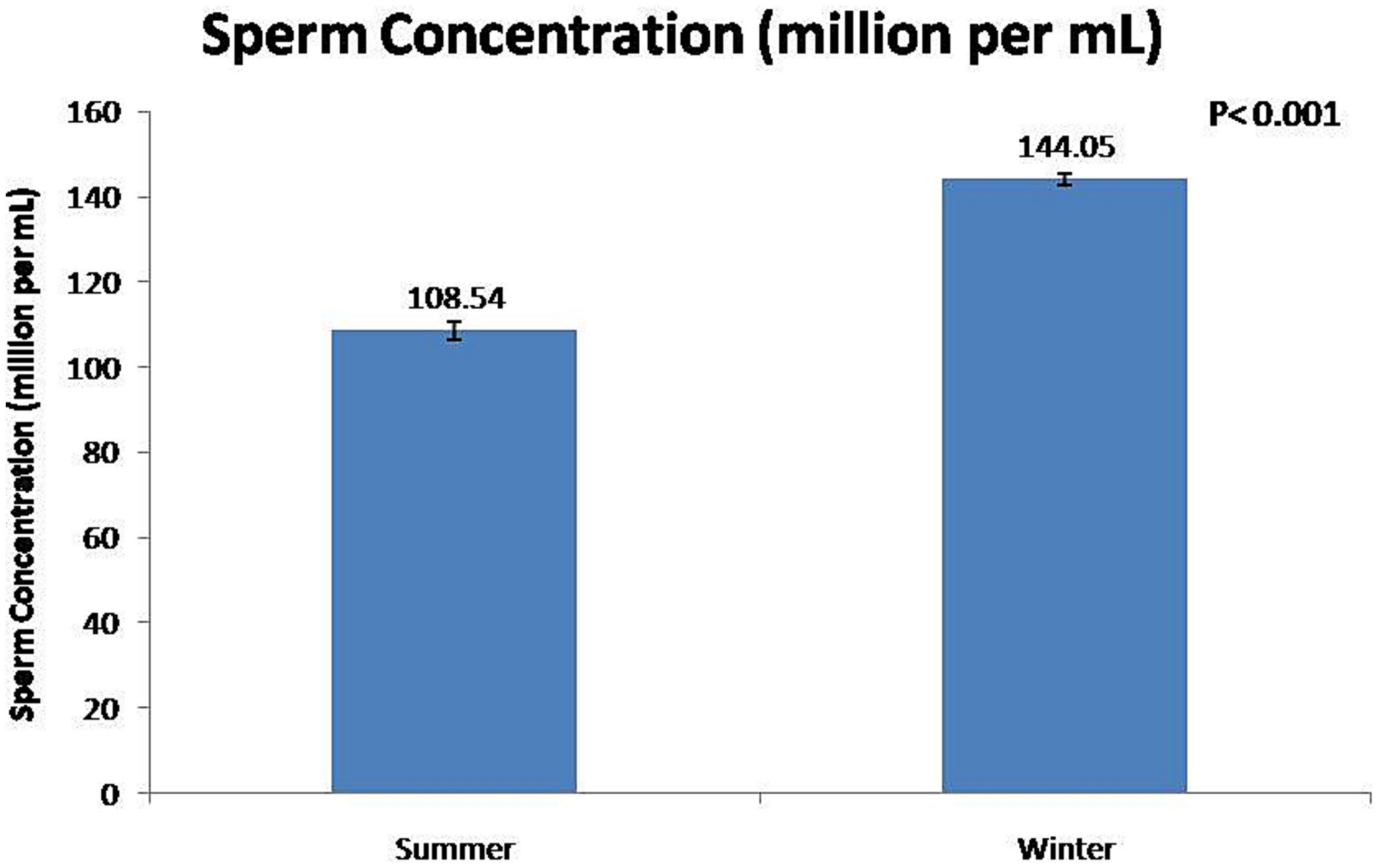
Effect of season on boar sperm concentration.

**Figure 5:**
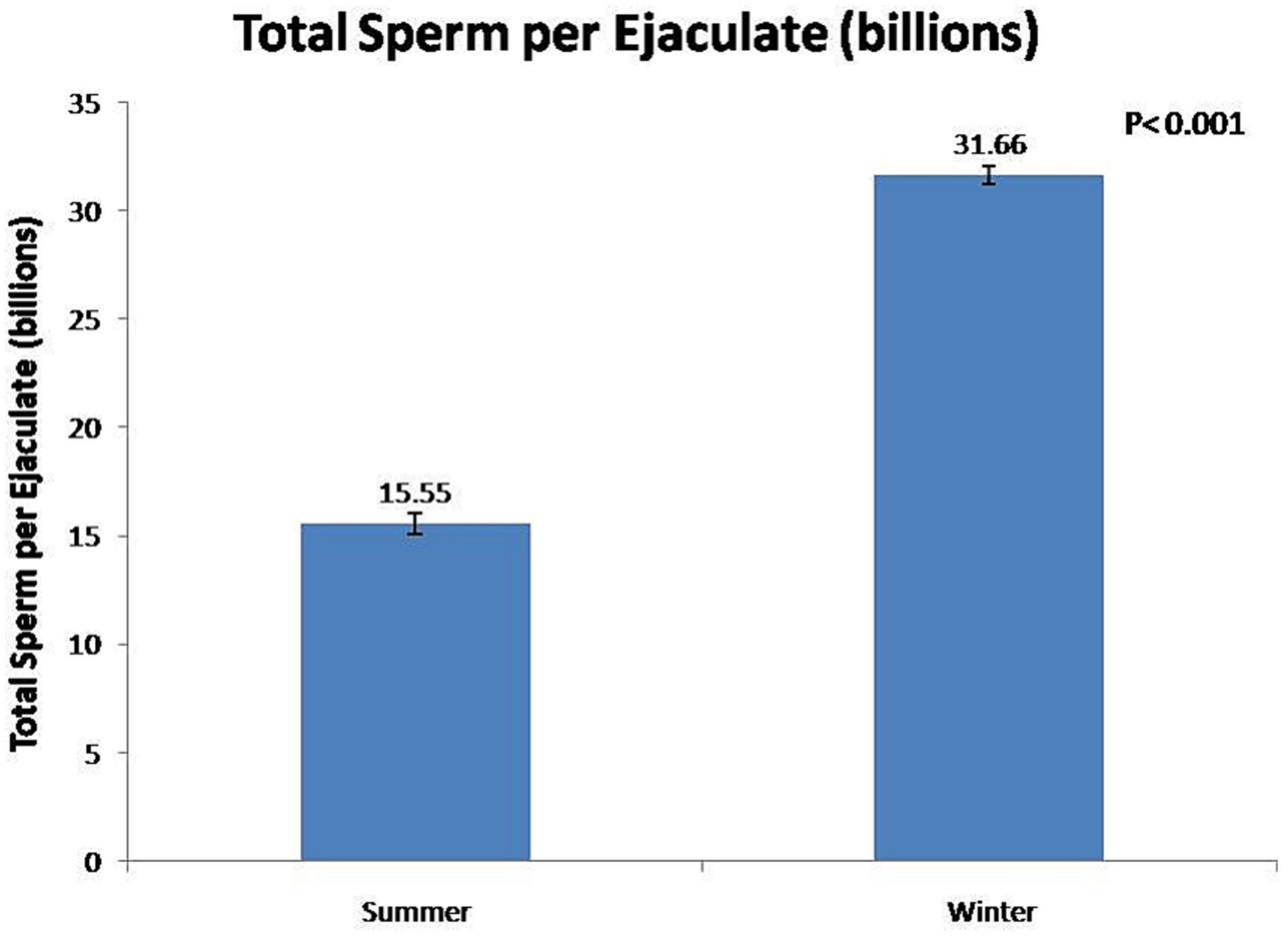
Effect of season on total sperm per ejaculate of boars.

At the fresh stage, season had a significant (*p* < 0.01) effect on boars’ sexual behavior and semen functional characteristics (Table 1). Summer season had a significant (*p* < 0.01) negative effect on reaction time and false mounts. In contrast, the winter season significant (*p* < 0.01) improved semen volume (Figure 3) and sperm concentration (Figure 4). In addition, total sperm per ejaculate (Figure 5) was significantly (*p* < 0.01) higher in the winter season. Sperm abnormality was significantly (*p* < 0.01) decreased in winter. SQPs, namely total motility, progressive motility, viability, acrosomal integrity, and HOST-reactive sperm, were significantly (*p* < 0.01) improved in the winter season. The following sperm kinematics parameters were significantly (*p* < 0.05) affected by season: VAP, VSL, VCL, ALH, BCF, and STR (Table 2); however, LIN did not differ significantly (*p* > 0.05) between the two seasons.

**Table 1:** Effect of season on boars’ sperm characteristics at the fresh stage in a subtropical climate (means ± SEM).

Parameters	Summer	Winter	*p*-value
Reaction time (min)	4.17 ± 0. 09^A^	2.15 ± 0.09^B^	<0.001
False mount (numbers)	2.02 ± 0.04^A^	1.41 ± 0.01^A^	<0.001
Sperm total motility (%)	76.51 ± 0.37^B^	89.82 ± 0.54^A^	<0.001
Sperm progressive motility (%)	30.21 ± 0.31	36.53 ± 0.29	<0.001
Viability (%)	81.40 ± 0.31^B^	90.29 ± 0.35^A^	<0.001
Abnormality (%)	12.71 ± 0.13^A^	7.47 ± 0.18^B^	<0.001
Acrosomal integrity (%)	79.07 ± 0.30^B^	87.34 ± 0.34^A^	<0.001
Hypo-osmotic swelling test (%)	76.33 ± 0.28^B^	84.76 ± 0.34^A^	<0.001

*n* = 84 ejaculates in each season; six ejaculates per boar. ^AB^Means with different superscripts in a row differ significantly (*p* < 0.05).

**Table 2:** Effect of season on boars’ sperm kinematics at the fresh stage in a subtropical climate (means ± SEM).

Parameters	Summer	Winter	*p*-value
VAP (μm/s)	55.75 ± 0.18^B^	62.64 ± 0.28^A^	<0.01
VSL (μm/s)	44.89 ± 0.17^B^	48.79 ± 0.30^A^	<0.01
VCL (μm/s)	119.60 ± 0.48^B^	126.11 ± 1.26^A^	0.012
ALH (μm)	5.15 ± 0.02^B^	5.79 ± 0.01^A^	<0.01
BCF (Hz)	23.82 ± 0.20^B^	27.36 ± 0.21^A^	<0.01
STR (%)	0.80 ± 0.00^A^	0.78 ± 0.00^B^	<0.01
LIN (%)	0.37 ± 0.00^A^	0.39 ± 0.031^A^	0.14

*n* = 84 ejaculates in each season; six ejaculates per boar. ^AB^Means with different superscripts in a row differ significantly (*p* < 0.05).

### Boars’ sperm quality characteristics after 72 h of cold storage

Semen quality characteristics after 72 h of cold storage were significantly (*p* < 0.01) improved in the winter than in the summer season (Table 3). Similarly, there was a significant (*p* < 0.01) effect of season on the kinematics parameters VAP, VCL, ALH, BCF, and LIN (Table 4).

**Table 3:** Effect of season on boars’ sperm characteristics after 72 h of storage at 17 °C in a subtropical climate (means ± SEM).

Parameters	Summer	Winter	*p*-value
Sperm total motility (%)	64.91 ± 0.25^B^	73.45 ± 0.32^A^	<0.01
Sperm progressive motility (%)	25.11 ± 0.15^B^	31.85 ± 0.23^A^	<0.01
Viability (%)	72.08 ± 0.29^B^	77.17 ± 0.38^A^	<0.01
Abnormality (%)	17.85 ± 0.32^Ab^	11.57 ± 0.17^B^	<0.01
Acrosomal integrity (%)	68.19 ± 0.26^B^	74.44 ± 0.33^A^	<0.01
Hypo-osmotic swelling test (%)	66.82 ± 0.24^B^	73.41 ± 0.25^A^	<0.01

*n* = 84 ejaculates in each season; six ejaculates per boar. ^AB^Means with different superscripts in a row differ significantly (*p* < 0.05).

**Table 4:** Effect of season on boars’ sperm kinematics after 72 h of cold storage at 17°C in a subtropical climate (means ± SEM).

Parameters	Summer	Winter	*p*-value
VAP (μm/s)	52.92 ± 0.22^B^	57.79 ± 0.43^A^	<0.01
VSL (μm/s)	42.04 ± 0.23^A^	45.15 ± 0.33^A^	<0.01
VCL (μm/s)	109.45 ± 0.63^B^	122.91 ± 0.53^A^	<0.01
ALH (μm)	4.80 ± 0.01^B^	5.16 ± 0.01^A^	<0.01
BCF (Hz)	21.65 ± 0.15^B^	24.19 ± 0.18^A^	<0.01
STR	0.79 ± 0.00^B^	0.78 ± 0.00^A^	0.09
LIN	0.38 ± 0.00^A^	0.36 ± 0.00^B^	<0.01

*n* = 84 ejaculates in each season; six ejaculates per boar. ^AB^Means with different superscripts in a row differ significantly (*p* < 0.05).

### Sperm mitochondrial membrane potential

**Figure 6:**
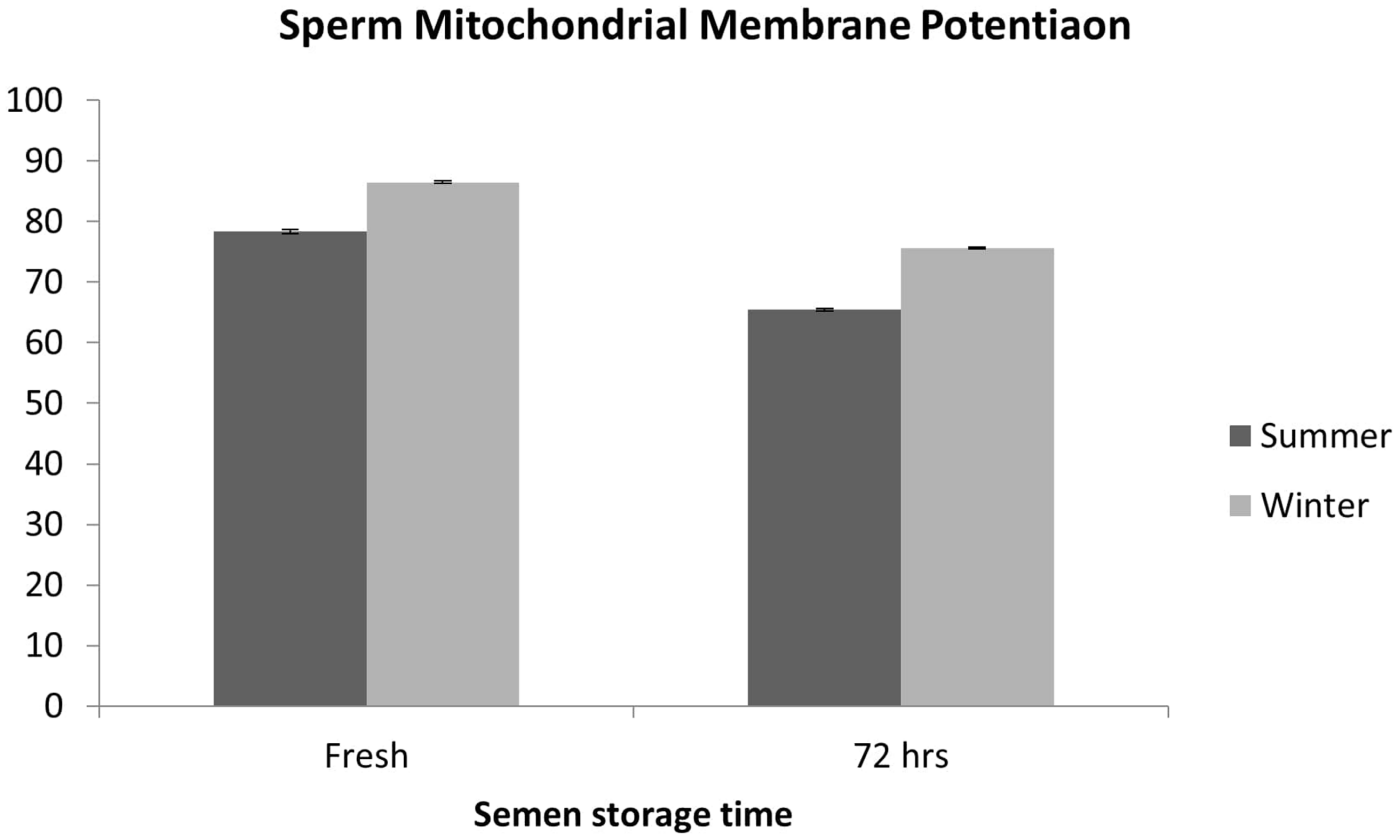
Effect of season on mitochondrial membrane potential of boar sperm.

Sperm MMP was severely compromised (*p* < 0.01) during summer both at the fresh stage and after 72 h of storage compared with the winter season (Figure 6).

### Boars’ seminal plasma antioxidant biomarkers

Seminal plasma TAC was significantly (*p* < 0.01) low in the winter season (Table 5). Seminal plasma MDA concentration was significantly (*p* < 0.01) increased during the summer season. Furthermore, seminal plasma GPx and CAT were significantly (*p* < 0.051) affected by season.

**Table 5:** Effect of season on boars’ seminal plasma antioxidant biomarkers in a subtropical climate (means ± SEM).

Parameters	Summer	Winter	*p*-value
TAC (mmol/L)	0.37 ± 0.00^B^	0.42 ± 0.00^A^	<0.01
MDA (nmol/mL)	1.87 ± 0.00^A^	1.72 ± 0.00^B^	<0.01
GPx (nmol/min/mL)	78.48 ± 0.49^B^	104.5 ± 0.75^A^	<0.01
CAT (nmol/min/mL)	4.91 ± 0.03^B^	6.81 ± 0.02^A^	<0.01

*n* = 84 ejaculates in each season; six ejaculates per boar. ^AB^Means with different superscripts in a row differ significantly (*p* < 0.05). TAC, total antioxidant capacity; MDA, malondialdehyde; GPx, glutathione peroxidase; CAT, catalase.

### *In vivo* fertility assessment

FR was significantly (*p* < 0.01) higher in the winter than in the summer season (Table 6). Similarly, LSB and LSW were significantly (*p* < 0.01) higher in the winter season, both of them being higher by three piglets than those in the summer season.

**Table 6:** Effect of season on boars’ *in vivo* fertility in a subtropical climate.

Season	*n*	Farrowing rate* %	Litter size at birth (LSB)	Litter size at weaning (LSW)
Summer	164	59.75^B^ (98/164)	8.18 ± 0.14^B^	7.46 ± 0.12^A^
Winter	181	78.45^A^ (142/181)	11.50 ± 0.12^A^	10.29 ± 0.09^B^
*p*-value		<0.01	<0.01	<0.01

Means with different superscripts in a column denote significant differences (*p* < 0.01). *n* = 164 multiparous sows in the summer season and 181 multiparous sows in the winter season. *Numerals enclosed within parentheses denote numbers.

## Discussion

In the present study, seasonal variations in semen quality and fertility of Hampshire–Ghungroo crossbred boars were investigated in a subtropical climate. In general, summer season had significant adverse effects on semen quality and fertility. Previous studies have documented season-dependent changes in boar semen quality in primary pork-producing countries with a temperate climate (7, 9, 26–30). To the best of our knowledge, this is the first study to document the effects of season on SQPs, sperm kinematics, MMP, antioxidant biomarkers, and *in vivo* fertility of Hampshire–Ghungroo crossbred boars in a subtropical climate.

Boars’ sexual behavior in terms of reaction time and false mounts was significantly better in winter than in summer. Consistent with our findings, similar results have been reported on boars reared in a high ambient temperature in China (27). In addition, heat stress has been shown to result in a decline in serum testosterone and estradiol concentrations in boars, thereby leading to reduced libido (27, 31). Similarly, a study has reviewed the effect of heat stress in boars in Thailand and reported that, during heat stress, crude protein intake is reduced, along with an increase in scrotal temperature, which may lead to poor libido (32). In boars, heat stress tends to trigger germ cells to undergo apoptosis and downregulate the StAR gene, which, in turn, affects the ability to reproduce and sexual behavior (16).

In the present study, season had a significant effect on ejaculate volume, sperm concentration, and total sperm per ejaculate. In the winter season, ejaculate volume, sperm concentration, and total sperm per ejaculate were increased by 52, 33, and 106%, respectively, compared with the summer season. The findings of this study show that the low sperm count per ejaculate during the summer season is a significant constraint to the optimal utilization of high-genetic-value boars in regions with a subtropical climate. It has been reported that, during the autumn–winter period in areas with a temperate climate, season rather than age had a significant effect on semen volume and sperm concentration (7, 28, 29, 33). In the summer season, Polish Large White and Duroc × Pietrain boars were least resistant to heat stress, and their semen volume and sperm concentration decreased during summer (12, 34). The higher total sperm output in boar ejaculate in winter was attributable to favorable ambient temperature for spermatogenesis (12, 34). In addition, studies have shown that the higher semen volume during the autumn–winter period is attributable to the increased activity of the accessory sex glands (12, 27). However, another study has reported that there is no major influence of seasonality on sperm quality and enzymatic scavengers in the south subtropical region of Brazil (35).

Sperm functional parameters at the fresh stage and after 72 h of cold storage were significantly higher during the winter season, whereas sperm abnormality was higher during the summer season. Similarly, sperm kinematics parameters were significantly improved during the winter season both at the fresh stage and after 72 h of cold storage. Sperm MMP at the fresh stage was lower in the summer season, which further deteriorated after cold storage. In boars, heat stress affects sperm motility through the downregulation of mitochondrial activity and ATP synthesis yield, which involves dephosphorylation of GSK3α and interference of mitochondrial remodeling (36). Previous studies have shown that heat stress induces oxidative stress, which further damages the mitochondrial function (37). Sperm progressive motility is strictly related to MMP and, as a consequence, to mitochondrial functionality and sperm fertility (38). The better sperm quality and sperm kinematics parameters observed during the winter season allow for the optimal utilization of high-genetic-value boars, whereas their efficient utilization in regions with a subtropical climate is restricted in the summer season. Similarly, previous studies have documented that sperm functional characteristics are significantly declined in the summer or during the long photoperiod and improved in the autumn–winter season (7, 33, 34, 39–42). Boars’ fertility decreases in summer due to heat-stress-induced poor sperm quality (29, 33). Furthermore, boars subjected to prolonged exposure to high temperatures have shown increased effects of heat stress on their semen quality (43). Studies have reported that boar ejaculates collected in spring and summer have a higher number of sperm head, tail, and acrosome defects (34, 44). In another study, high semen volume, high sperm concentration, and high sperm motility were recorded in the autumn–winter season (45). Season-dependent irreversible changes in sperm morphology have been reported in boar semen during cold storage at 17°C (34). Boar sperm collected in the summer season are more prone to cold storage damage than those collected in winter because of the differences in temperature and photoperiod. In AI programs using liquid boar semen, the effect of season on semen quality should be taken into account. Sperm quality analysis, including sperm cell structures, should be routinely carried out at boar semen stations, particularly during the summer season (34). It is well known that boar semen quality deteriorates during liquid storage; however, it deteriorates more rapidly during summer. In summer, the higher ambient temperature has a negative effect on spermatogenesis, which may result in increased sperm abnormalities. Sperm head defects have been associated with chromatin damage in the cell nucleus during spermatogenesis (46, 47). Moreover, high ambient temperatures have an adverse effect on the thermoregulation of boars’ testes, which may enhance sperm abnormalities (40). Boars are sensitive to heat stress because of their body physiology, which, in turn, affects their reproductive efficiency (1, 48, 49). Heat stress has negative effects at every stage of the spermatogenesis process, and it may damage sperm DNA (50, 51). Consequently, sperm morphological abnormalities due to heat stress may lead to poor sperm functional competence and decreased boar fertility (52, 53).

In boars, sperm membrane plays an important role in fertilization, and it is used as an indicator of sperm health. It has a high polyunsaturated fatty acid, high phospholipid, and low cholesterol content, and therefore, it is susceptible to oxidative damage (54). During liquid storage, boar sperm are prone to oxidative damage due to higher ROS production and decreased performance of the natural antioxidant defense system (6, 55, 56). A low concentration of ROS is required for normal sperm function, whereas its high concentrations have deleterious effects, damage sperm DNA, inhibit sperm–oocyte fusion, and reduce sperm motility (57). The findings of the present study revealed a significant effect of season on boar semen antioxidant biomarkers. In the summer season, antioxidant capacity was significantly compromised and lipid peroxidation was significantly increased compared with the winter season. These findings partly explain the poor quality of boar semen during the summer season. Increased ROS production in summer affects sperm quality and fertility. It also affects spermatogenesis, sperm maturation, mitochondria function, sperm membrane, and sperm DNA. In line with the findings of the present study, a previous study has reported that dietary L-arginine supplementation improves semen TAC and GPx and CAT activities in boar semen (27). High heat stress might lead to supraphysiologic ROS production, which may compromise the structural integrity and functional competence of sperm (58), such as peroxidative damage to the sperm plasma membrane and DNA strand breakage in the sperm nucleus (59). It has been previously reported that oxidative stress damages the integrity of sperm DNA (60). In tropical summer, heat stress induces DNA fragmentation in boar spermatozoa (61). For optimal spermatogenesis, it is recommended that the testicular temperature of boars be maintained 4°C to 6°C lower than core body temperature (62). However, high ambient temperatures may increase testicular temperature, which has a detrimental effect on spermatogenesis and the resultant spermatozoa. Therefore, thermoregulatory failure due to heat stress can compromise the functional competence and fertility of sperm (7, 27, 51).

Achieving viable pregnancies and a reasonable litter size with healthy piglets after *in vivo* insemination is the most important indicator of a boar’s fertility. In the present investigation, season had a significant effect on the *in vivo* fertility of boars. FR, LSB, and LSW decreased significantly in summer compared with winter. A significant difference of three piglets in LSW and LSB was observed between the summer and winter seasons. These findings demonstrate that boar semen fertility is severely compromised during hot and humid summer months in regions with a subtropical climate. In agreement with the findings of the present experiment, previous studies have documented that increased ambient temperature above the thermal neutral zone results in a decline in boar fertility in summer months because of the associated metabolic changes (4–6). Heat stress affects the reproductive function of boars via germ cell apoptosis and downregulation of the StAR gene (16). Boars exposed to temperatures higher than the ambient temperature for prolonged periods have shown poor sperm quality and reduced fertility compared to boars maintained at 23°C (63). In summer, linseed oil supplementation improved boars’ *in vivo* fertility compared to the control group (10). In tropical and subtropical regions, high temperature and humidity adversely affect boars’ reproductive efficiency (61). Mean fertility in the summer season is lower (81.2%) than in winter (86.8%) in regions with a temperate climate (64). In Australia, FR during the summer–autumn season is 77.1% compared with 91.9% in spring (65). Similarly, in the Philippines, after exposure to higher ambient temperatures, FR, LSB, and LSW of boars decreased significantly (66). Recently, a higher FR has been reported in sows inseminated during the hot and humid season in a region with subtropical climate (67).

## Conclusion

The findings of the present investigation offer significant insights into the effects of season on reproduction in crossbred boars attributable to heat stress in a region with a subtropical climate. In the summer season, lower sperm MMP and downregulation of antioxidant biomarkers resulted in a significant decline in semen volume, sperm concentration, sperm quality, and sperm kinematics of Hampshire–Ghungroo crossbred boars in regions with a subtropical climate. Total sperm per ejaculate was higher in the winter season, which allows more efficient utilization of breeding boars in AI programs. *In vivo* fertility was also significantly reduced during the summer season compared with the winter season due to heat stress. As environmental temperature and humidity are extremely high in regions with a subtropical climate, necessary interventions in the form of genetics (less exotic inheritance), management, and nutrition are needed for the optimal utilization of breeding boars in these regions.

## Data Availability

The original contributions presented in the study are included in the article/supplementary material, further inquiries can be directed to the corresponding author.
